# Differential Packing of Cs_2_Mo_6_Br_14_ Cluster-Based Halide in Variable Diameter Carbon Nanotubes with Elimination and Polymerization to 1D [Mo_2_Br_6_]_*x*_ Ising Model Structures by Steric Confinement

**DOI:** 10.1021/jacs.4c14883

**Published:** 2025-02-19

**Authors:** Eric Faulques, Victor G. Ivanov, Stéphane Cordier, Reza J. Kashtiban, Yann Molard, Jean-Luc Duvail, Nataliya Kalashnyk, Jeremy Sloan

**Affiliations:** † Univ. Lille, CNRS, Centrale Lille, Univ. Polytechnique Hauts-de-France, UMR 8520-IEMN, F-59000 Lille, France; ‡ Sofia University, St. Kliment Ohridsky, Faculty of Physics, 5 James Bourchier Boulevard, 1164 Sofia, Bulgaria; § Univ Rennes, CNRS, ISCR, Institut des Sciences Chimiques de Rennes−UMR6226, F-35000 Rennes, France; ∥ Department of Physics, University of Warwick, Coventry CV4 7AL, U.K.; ⊥ Nantes Université, CNRS, Institut des Matériaux de Nantes Jean Rouxel, IMN, F-44000 Nantes, France

## Abstract

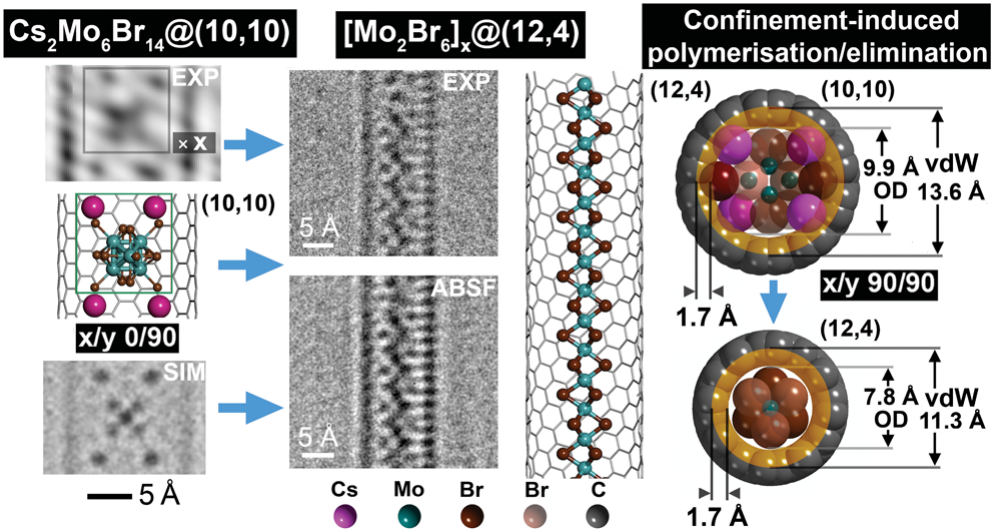

We present detailed findings on the imaging, structure, and vibrational properties of novel hybrids of the red-emitting octahedral cluster-based compound Cs_2_Mo_6_Br_14_ encapsulated within single-walled carbon nanotubes (SWCNTs) of varying diameter. We explore the subtle relationship between the SWCNT internal diameter and Cs_2_Mo_6_Br_14_ cluster packing and find a hierarchical relationship between the nature of the cluster packing and a progressive tendency toward formation of one-dimensional (1D) structures as the SWCNT diameter narrows from 24 to 11 Å. As the internal SWCNT van der Waals radius approaches the outside diameter (OD) of the [{Mo_6_^II^Br_8_^i^}Br_6_^a^]^2–^ (more simplistically, [Mo_6_^III^Br_14_]^2–^) molecular anion species, SWCNT steric confinement causes a compositional elimination and polymerization resulting in the formation of reduced extended [Mo_2_^III^Br_6_]_*x*_ nanoribbons which approximate 1D Ising model structures. Our experimental results, obtained through high-resolution transmission electron microscopy and Raman spectroscopy, are supplemented by density functional theory (DFT) calculations.

## 1 Introduction

A fuller understanding of the role of single-walled carbon nanotubes (SWCNTs) as templates for the self-assembly of functionalized architectures at the nanoscale is crucial for their future applications. SWCNTs can be filled with many types of crystals, including metal and halogen ions,^1−3^ single element^4−6^ and binary atomic chains,^7,8^ atomic coils,^2,9^ twisting halide chains,^10^ transition metal dichalcogenide and monochalcogenide nanowires,^11−14^ chains of organic/inorganic molecules,^15−20^ and, most recently, subnanometer wide halide perovskite nanowires.^21,22^ The encapsulation of such atomically limited scale species provides valuable insights in terms of their confined structural properties but also allows access to the electronic characteristics of such hybrids and, in special cases, profound adjustments to the local chemistry, including changes in stoichiometry and oxidation state, of the encapsulated species due to strong steric confinement.

Our preliminary objective was to fill or decorate SWCNTs with phosphorescent species, which have been effective in modifying the optical properties of nanoparticles, porous materials, copolymers, and quantum dots.^23,24^ Research so far on decorating or filling single-walled carbon nanotubes (SWCNTs) with chromophores or luminescent substances has been limited but has nonetheless demonstrated some important findings.^25−27^ Synthesizing SWCNT hybrids with filling compounds fluorescent in the near-infrared range (NIR) of the optical spectrum (∼700–800 nm)^27^ is also promising for telecommunications with optical fibers in order to bring the photoemission of CNTs to within the silica transparency window. By using NIR/short-wavelength infrared (SWIR) emitters based on composites with carbon nanotubes, it may be possible to tune the photoluminescence in this window to select specific wavelengths to transfer information. Emission at 1530–1565 nm (C band) used for conventional telecommunications^28^ is possible by semiconducting SWCNTs, as well as all O- to U-telecom bands (i.e., 1260–1675 nm).

We have therefore modified SWCNTs by incorporating NIR phosphorescent transition metal nanocluster-based compounds, such as Cs_2_Mo_6_X_14_ (X = Cl, Br, I),^29−37^ in them. Recall that Cs_2_Mo_6_Br_14_ is a ternary halide synthesized at high temperature whose crystal structure is built up from [{Mo_6_Br_8_^i^}Br_6_^a^]^2–^ anionic building blocks (Br^i^ for inner ligand and Br^a^ for apical ligand) and Cs^+^ cations.^37^ The utilization of metal atom clusters in nanotubes can potentially be an added value for biophysical applications, as these exhibit a quantum yield of >20%. Such modified nanotubes may therefore show great potential for selectively tagging specific predefined cancer targets or other diseases.^38^ Moreover, they could operate within the transparency window of biological tissues, offering multiple emission wavelengths between 700 and 800 nm for the clusters and 1.2–2 μm for the nanotubes. In addition, the intrinsic photoluminescence (PL) of SWCNTs is highly sensitive to their environment, making them ideal for the development of SWCNT-based biological and chemical sensors. Notably, both unfilled and filled SWCNTs are more photostable compared to other labeling substances such as Nile blue or Nile red, a significant advantage. Lastly, SWCNTs and their hybrids offer a substantial contact area in the investigated target samples due to their immense aspect ratio (ranging from 500 to approximately 10^8^), which further facilitates potential applications as described above.

This report presents evidence of SWCNT filling with the NIR phosphorescent cluster-based halide Cs_2_Mo_6_Br_14_,^39,40^ by harnessing high-resolution transmission electron microscopy (HRTEM) images of individual filled SWCNTs and Raman spectroscopy of the hybrid composite material. An intriguing observation is that the cluster moieties either exhibit a novel organization or undergo transformations within the nanotubes, resulting in distinctive molecular or polymeric structures closely regulated by the encapsulating SWCNT diameter. The spectroscopy studies reveal that cluster encapsulation significantly modifies the vibrational and electronic properties of both the original cluster and the encapsulating SWCNT. The findings suggest that the chosen melting method for infiltration enables the successful encapsulation of luminescent clusters within wider carbon nanotubes while maintaining their integrity. However, our results also indicate that structural transformation of the chosen cluster compound occurs readily within narrow single-walled carbon nanotubes. Through the utilization of molecular and periodic models, density functional theory (DFT) calculations on isolated ions and one-dimensional filling crystals corroborate experimental observations by HRTEM and Raman spectroscopy.

## 2 Results and Discussion

### 2.1 HRTEM Images

**Figure 1:**
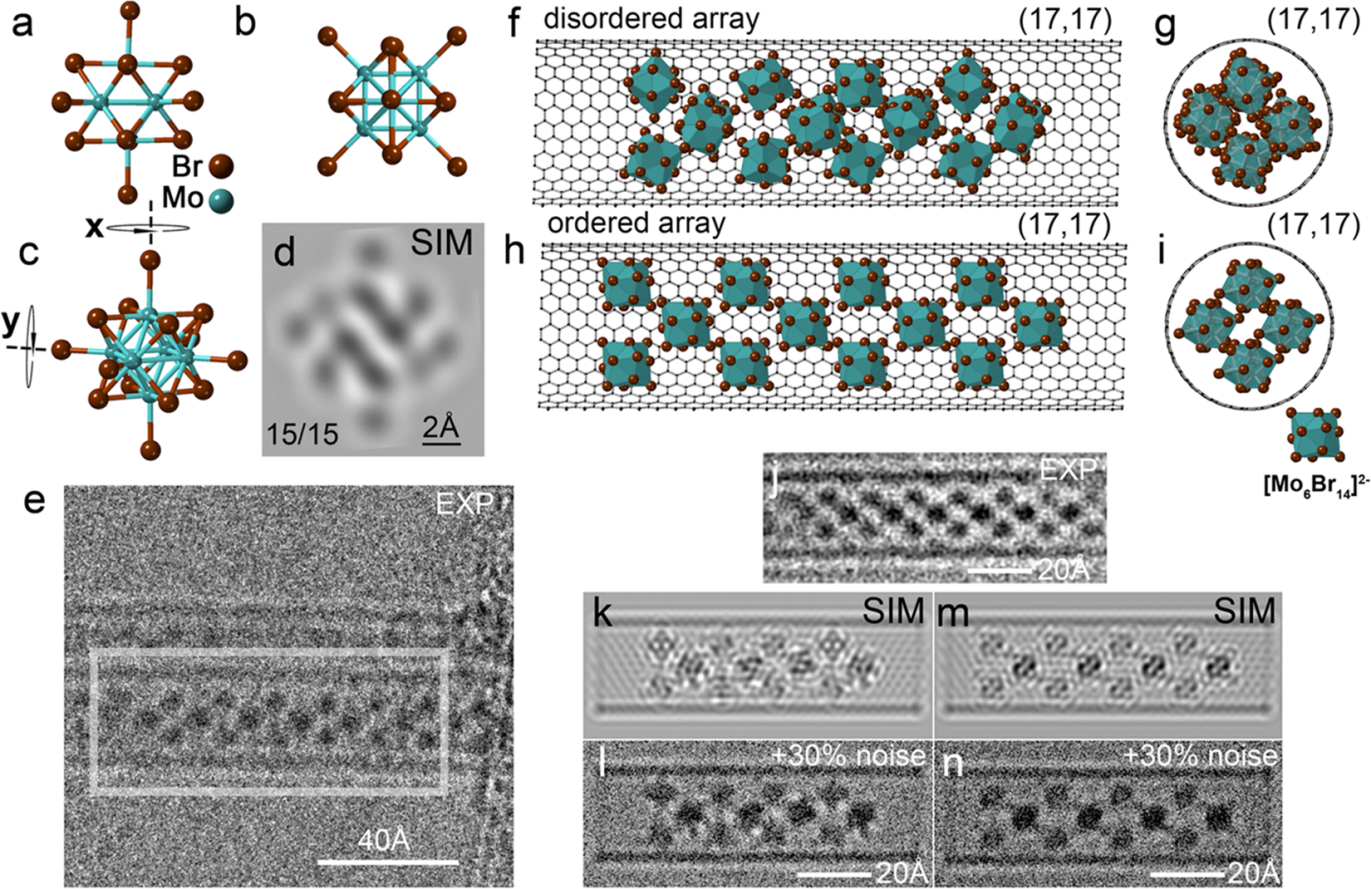
(a) [Mo_6_Br_14_]^2–^ anion viewed along the 2-fold axis (i.e., *x*/*y* = 0/0, 90/0, or 90/90, Figure 2) which is chosen as the arbitrary starting orientation for the simulation tableau in Figure 2. (b) The [Mo_6_Br_14_]^2–^ anion viewed along a 4-fold axis (i.e., *x*/*y* = 45/0 or 0/90, Figure 2). (c) Perspective view of the [Mo_6_Br_14_]^2–^ anion (e.g., *x*/*y* = 15/15) showing the *x* and *y* rotation axes used in the simulation tableau (Figure 2). (d) Multislice simulation of the anion in (c) with this orientation. (e) Experimental image of [Mo_6_Br_14_]^2–^ anions packed into a ∼23 Å diameter SWCNT. (f, g) Schematic side (left) and end-on depiction (right) of a partially disordered array of [Mo_6_Br_14_]^2–^ anions, with each ion randomly rotated by 3° (i.e., relative to *x*/*y* = 30/15, Figure 2) packed into a (17,17) SWCNT. (h, i) as for (f, g) but with an ordered array of [Mo_6_Br_14_]^2–^ anions (i.e., all ions have the same *x*/*y* = 30/15 orientation). (j) Detail from the indicated region in (e). (k, m) Multislice simulations from models in (f, h) respectively. (l, n) as (k, m) but with 30% random noise added.

Observations of the pristine, unfilled SWCNTs indicate that more than 90% of SWCNT samples have mean diameters ranging from 10 to 23 Å, with the remaining tubes typically deviating by a maximum of 5 Å from this range. Images taken after treating SWCNTs with clusters using the described protocol provide evidence of a high fraction of successfully encapsulated samples with various crystalline structures or molecular arrangements inside the tubes. Figures 1 and 3–5 show HRTEM images of NanoIntegris (NI) and SouthWest NanoTechnologies (SWeNT) SWCNTs filled with Cs_2_Mo_6_Br_14_. The nature of the packing of [Mo_6_Br_14_]^2–^ anions is worth further exposition, given the relationship with the SWCNT diameter, which exists in the median 13 to 17 Å range for NI96 SWCNTs and in the reduced median 7–11 Å diameter range for SWeNT SWCNTs. As we report below, however, only SWCNTs with diameters of ∼11 Å and above show significant filling. In general, we find instances of loosely bound clusters in wider SWCNTs forming semiordered cluster arrays, in which they vibrate in position when imaged by HRTEM but, in the lower diameter limit, such clusters polymerize to form continuous [Mo_2_Br_6_]_*x*_ chains accompanied by a possible transmutation/elimination of CsBr, Mo_3_Br_6_, and Mo^0^ to maintain chemical balance overall in the sample.

Figure 1(a–c) shows various structural projections of the *O*_*h*_ symmetry [Mo_6_Br_14_]^2–^ anion along the 4-fold and 2-fold axes in perspective and with *x* and *y* rotation axes defined with respect to two orthogonal 4-fold axes. Figure 1(d) is a multislice HRTEM simulation^41^ obtained for optimum defocus for our HRTEM (Experimental Section). Figure 1(e) shows an experimental image of alternating 2 × 1 and 1 × 2 packed arrays of [Mo_6_Br_14_]^2–^ anions inside a ∼23 Å diameter SWCNT. Two views (i.e., side on and end on) of the [Mo_6_Br_14_]^2–^ anions are indicated in Figure 1(f,g), in which the [Mo_6_Br_14_]^2–^ anions are packed in random orientations within the SWCNT. A further possibility is presented in two views in Figure 1(h,i), in which the anions are more rigidly packed. In both sides of views in Figure 1(f,h), the [Mo_6_Br_14_]^2–^ anions are separated diagonally by ∼8 Å and then ∼12.5 Å longitudinally along the tube axis. In Figure 1(j), an average background subtraction filtered (ABSF)^42^ version of detail obtained from the indicated region in Figure 1(e) is presented. This we compare with optimum defocus multislice simulations both without (i.e., Figure 1(k,m)) and with 30% noise added (i.e., Figure 1(l,n)) to more closely represent the low-dose experimental imaging conditions. Visual comparison of the simulations indicates that a more rigid packing of the [Mo_6_Br_14_]^2–^ anions (i.e., Figure 1(h,i)) more closely reproduces the observed imaged cluster array (i.e., Figure 1(j)).

**Figure 2:**
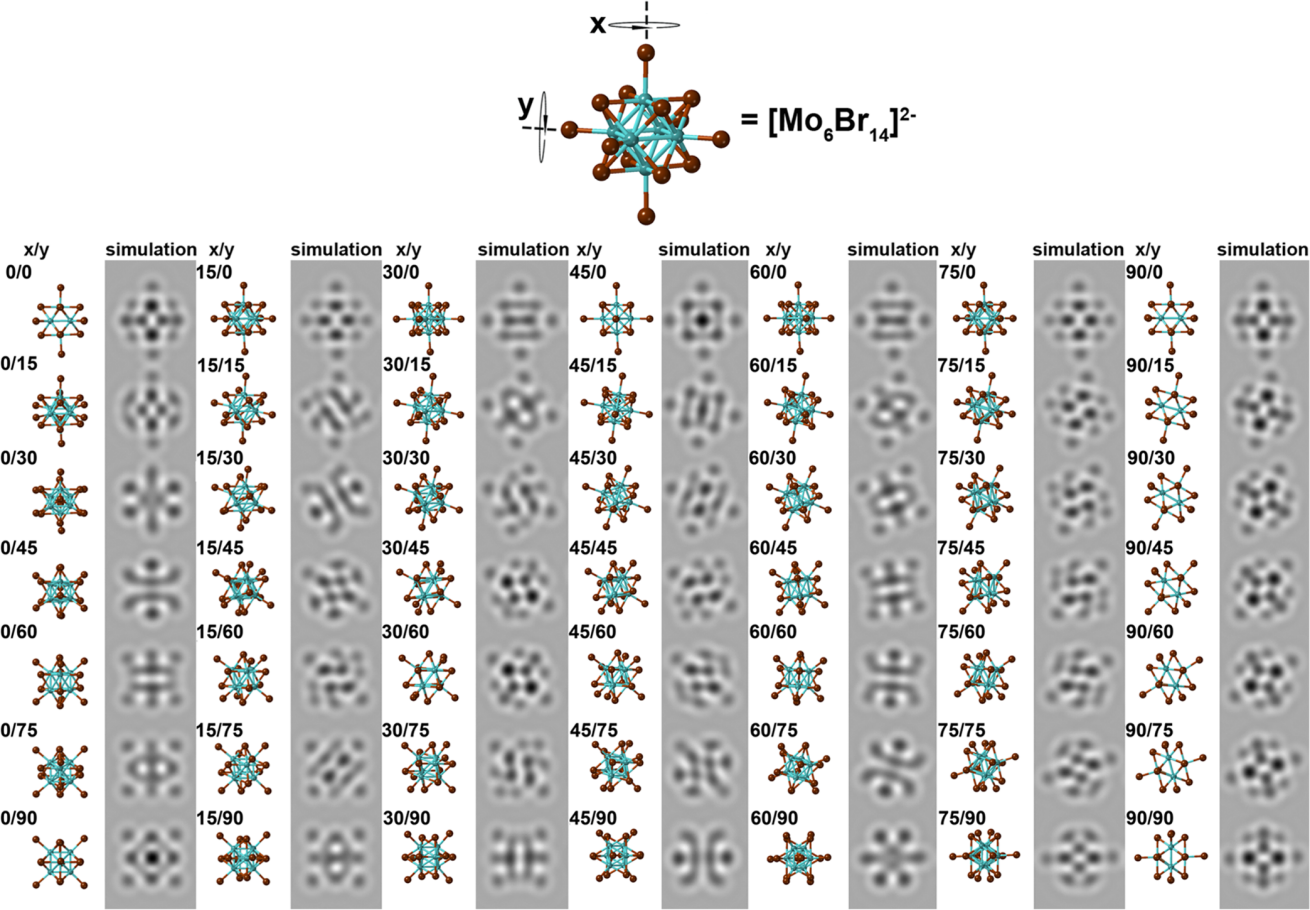
Tableau of [Mo_6_Br_14_]^2–^ structure models on the left in each column and multislice HRTEM image simulations on the right in each column calculated for the ideal defocus viewed in different *x*/*y* orientations (*x* and *y* axes defined in Figure 1 and in the model at top). This tableau includes only a partial subset of all possible orientations (i.e., from 0 to 90° about *x* and *y* in increments of 15°, as indicated) of the cluster anion; the remainder may be reproduced from mirror plane and rotational symmetry operations from individual ion orientations in the tableau. Cs atoms are omitted for clarity.

Figure 1(a–c) also reproduces three orientations from a rotation/simulation tableau, as presented in Figure 2. These tableaus can be used to identify or predict the orientation of inorganic molecular anions in nanotubes, such as the Lindqvist ion [W_6_O_19_]^2–^,^18,19^ which has similar *O*_*h*_ symmetry but which is not isostructural with either [Mo_6_Br_14_]^2–^ or [Mo_6_I_14_]^2–^ (also imaged within SWCNTs^20^) when they are static enough that their structure can be imaged by HRTEM. The [Mo_6_Br_14_]^2–^ orientations are defined in terms of 15° interval rotations about two orthogonal axes, *x* and *y*, as indicated in Figure 1(c). While the maximum rotation employed was 90°, the remaining 90°+ to 180° orientations can readily be derived from simple mirror plane and rotation symmetry operations as previously described.^18,20^

**Figure 3:**
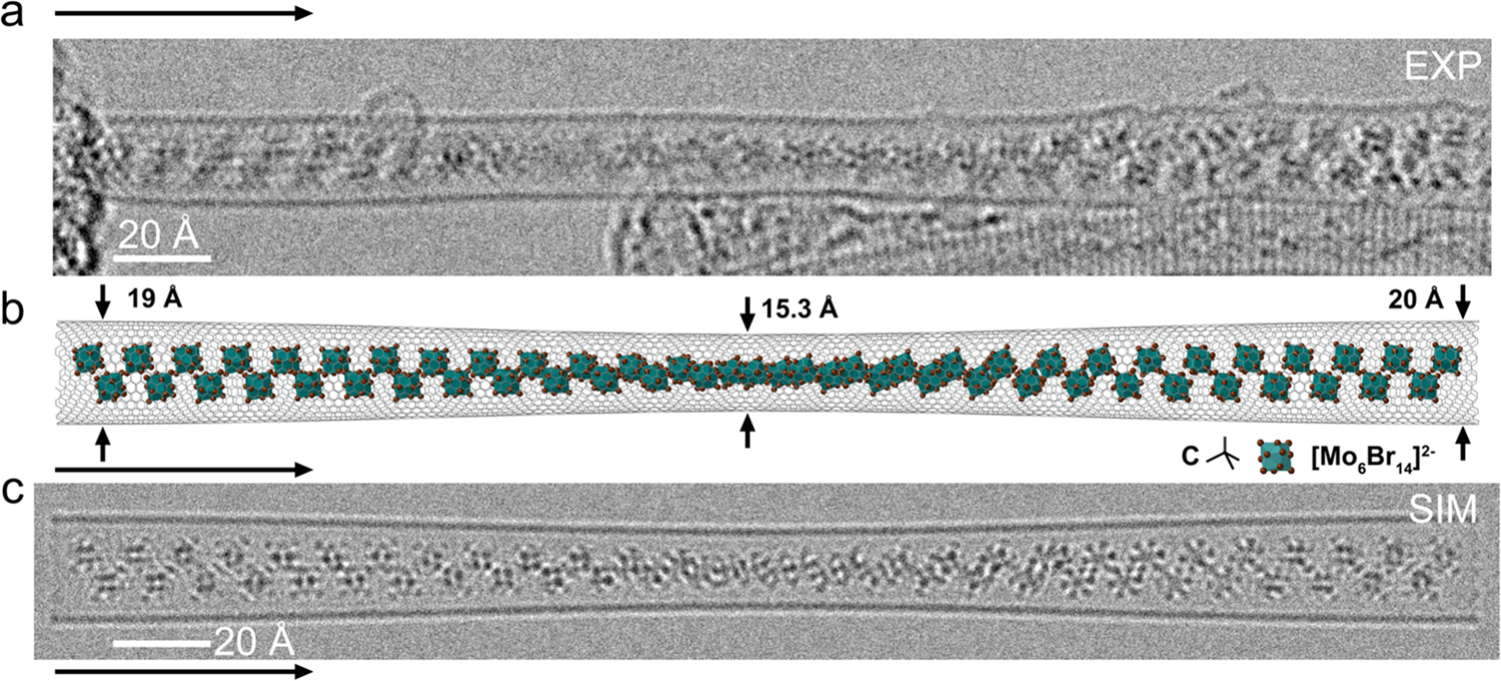
HRTEM Image of an ∼18 Å average diameter elliptically distorting SWCNT packed with a “zigzag” array of [Mo_6_Br_14_]^2–^ ions. The anion arrangement can most easily be visualized by viewing the composite at a glancing angle along the indicating arrow. (b) Schematic model of a “zigzag” array of [Mo_6_Br_14_]^2–^ ions derived from the arrangement in (a). Cs^+^ ions are omitted for clarity. (c) Multislice HRTEM image simulation produced at optimum defocus from the model in (b). As with (a–c), the helical arrangement of [Mo_6_Br_14_]^2–^ ions can best be visualized by viewing all three figures along a glancing angle with respect to the indicating arrows.

In Figure 3(a), an HRTEM image of a twisting nanowire consisting of a rotating 2 × 1 array of [Mo_6_Br_14_]^2–^ anions is presented. The asymmetric 2 × 1 arrangement of the anions in the cross-section causes an elliptical distortion in the encapsulating SWCNT, which can be visualized by viewing the composite at a glancing angle in the indicated direction. The elliptical distortion in the SWCNT causes an apparent variation in the diameter of the SWCNT measured in plan view from ∼15.3 Å at the narrowest point and 20 Å at the widest, as indicated in the approximate schematic model in Figure 3(b). The schematic model in Figure 3(b) is based on a 2 × 1 array of [Mo_6_Br_14_]^2–^ anions formed inside an elliptical (17,15) SWCNT with an aspect ratio of 1:1.3 (see also Figure 6(g) below). From this distorting model, we produced the multislice simulation in Figure 3(c), which schematically reproduces the contrast of the rotating 2 × 1 array of [Mo_6_Br_14_]^2–^ anions that we observe experimentally in Figure 3(a) concomitant with the elliptically distorting SWCNT. The “twisting effect” of the 2 × 1 array of [Mo_6_Br_14_]^2–^ anions can be compared in Figure 3(a–c) by viewing each along the indicating arrows.

**Figure 4:**
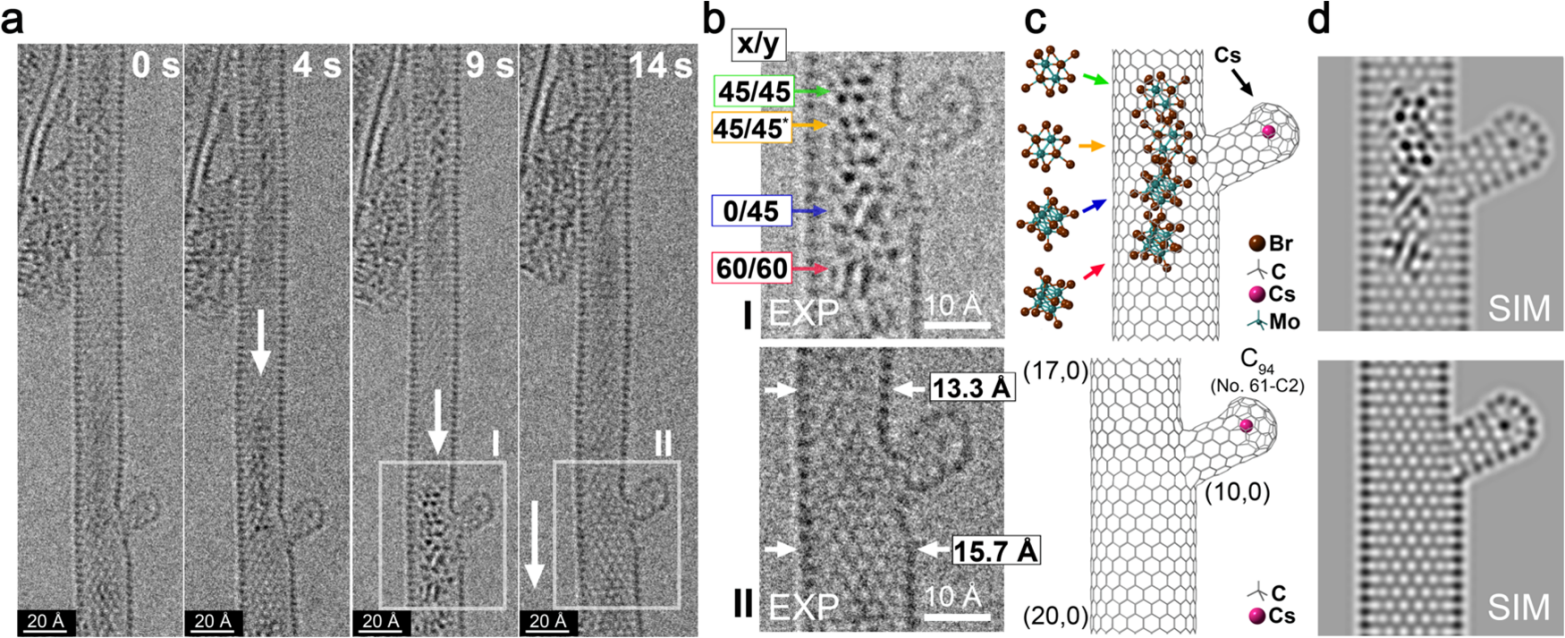
(a) Time-resolved sequence of a group of [Mo_6_Br_14_]^2–^ ions moving along a well-resolved “zigzag” (17,0) SWCNT with the images obtained at 0.8 s exposures for a total of 14 s. The group of [Mo_6_Br_14_]^2–^ ions can be viewed clearly at 9 s (i.e., region **I**) but disappears in the next region (i.e., region **II**). In this last image, the encapsulating defective regions of SWCNT can be clearly visualized. (b) Detail from region **I** (obtained at 9 s) in (a). At least four [Mo_6_Br_14_]^2–^ ions can be seen in the indicated *x*/*y* orientations as derived from the tilt tableau in Figure 2. Here and in Figure 4(c), the asterisk indicates anions with similar *x*/*y* orientations being rotated horizontally by some angle. In region **II** (obtained at 14 s), the impediment in the now-empty SWCNT can now be seen. (c) Schematic models derived from details **I** and **II**. The top model shows the arrangement of [Mo_6_Br_14_]^2–^ ions derived from **I** and with a single visible Cs^+^ atom indicated in the outcrop. The bottom model indicates the composite nanotube derived from detail **II**, which consists of a narrower (17,0) SWCNT interfacing with a wider (20,0) SWCNT with an outcrop at the interface comprising a short (10,0) SWCNT capped by a cap derived from half a C_94_ molecule. (d) Multislice simulations corresponding to the top and bottom models in (c), which well reproduces the contrast features for details **I** and **II** in (b).

All of the [Mo_6_Br_14_]^2–^ anions imaged in this study tended to undergo dynamic motion in the presence of an electron beam, often making it difficult to visualize them directly. We provide several examples of movies showing the motion of [Mo_6_Br_14_]^2–^ anions under the influence of the electron beam in the Supporting Information (Movies S1, S2, S3, and S4). Some exceptions to this behavior were observed when the ions were more tightly packed, as in the cases of Figures 1(e) and 3(a), although even in these instances, the individual ions were frequently blurred and difficult to see. As the SWCNT diameters narrow toward 15 Å, it became possible to see more structural detail in the anions, a process that could also be enhanced when the ions encountered unusual defects in the SWCNTs, which could trap groups of ions. In Figure 4(a), four time-resolved images, obtained over a period of 14 s, show motion of a group of 4–5 [Mo_6_Br_14_]^2–^ anions along a (17,0) SWCNT, which widens to a (20,0) SWCNT beyond a small outcrop consisting of a ∼(10,0) short SWCNT closed off by a cap (fashioned, in this case, from half a C_94_ fullerene molecule). At 9 s, the [Mo_6_Br_14_]^2–^ group is trapped (detail **I**), and we see at least four discrete anions in different *x*/*y* orientations (defined in Figure 2) as indicated in Figure 4(b) and the corresponding top model in Figure 4(c), which was constructed from the indicated SWCNT sections in the bottom model. In Figure 4(d), a multislice HRTEM simulation is presented based on the composite top model in Figure 4(c). After 14 s, this cluster group disappears (detail **II**), leaving an empty composite SWCNT as represented in the corresponding bottom HRTEM detail, model, and multislice simulation in Figure 4(b,c).

**Figure 5:**
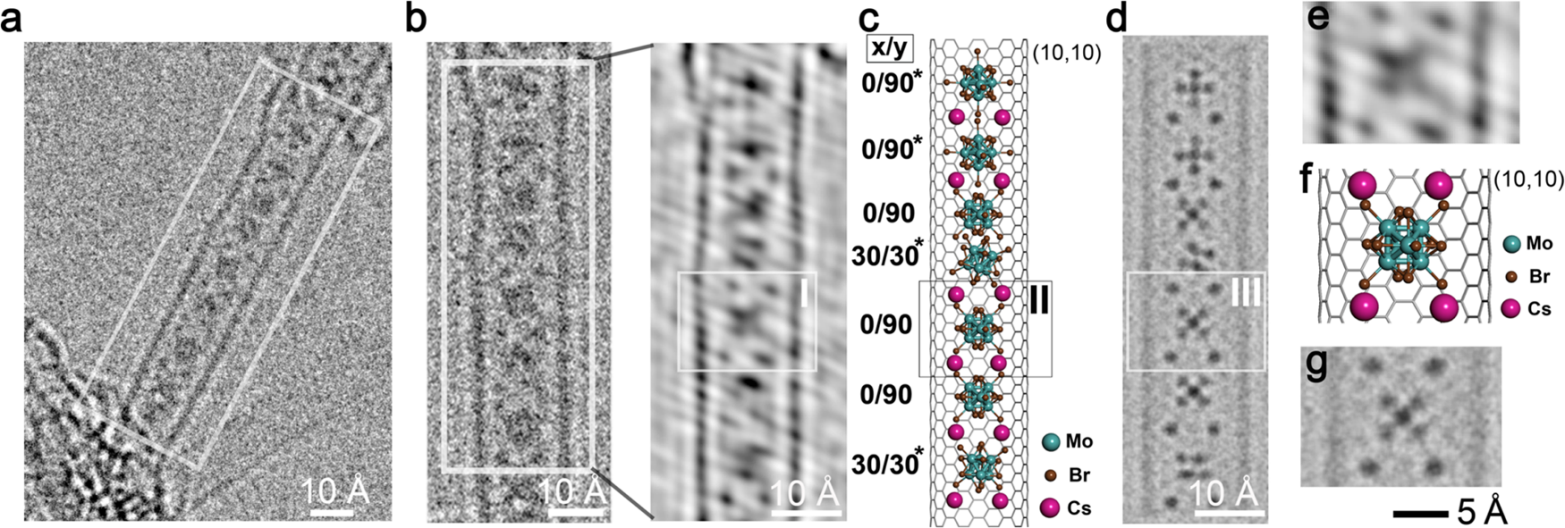
(a) Raw image of a ∼13.6 Å SWCNT filled with a linear chain of seven [Mo_6_Br_14_]^2–^ anions with some contrast due to the accompanying Cs^+^ visible. (b) Boxed region from (a) and ABSF filtered detail (right). The Cs^+^ cations are visible as clear black spots, the [Mo_6_Br_14_]^2–^ anions as blurs. The most clearly visible cation/cluster region is at **I**. (c) Derived model with the positions of the Cs^+^ cations indicated as well as the approximate position and *x*/*y* orientation of each anion as derived from the simulation tableau in Figure 2. (d) Multislice simulation obtained from the model in (c). (e–g) details from the indicated regions **I**, **II**, and **III** (i.e., in (b, d)), respectively.

In most of the HRTEM images that we obtained of the [Mo_6_Br_14_]^2–^ anions, it was only occasionally possible to see the counterbalancing Cs^+^ cations, of which there should normally be two per anion, due to their rapid dynamical motion of the latter in the electron beam. In narrow SWCNTs with diameters below ∼14 Å, it has been possible to image both the [Mo_6_Br_14_]^2–^ anions and the counterbalancing Cs^+^ cations. In Figure 5(a) and the corresponding details and ABSF version, the positions of both the [Mo_6_Br_14_]^2–^ anions and Cs^+^ cations with approximately the correct balance between both are visible. The contrast due to the [Mo_6_Br_14_]^2–^ anions is blurred, but the Cs^+^ cations appear in pairs of dark spots distributed evenly between the cations, although one Cs^+^ pair is missing. In Figure 5(c), a structural representation of the filtered structure in Figure 5(b) is given, indicating the relative orientation of the [Mo_6_Br_14_]^2–^ anions and disposition of the Cs^+^ counterions. In Figure 5(d), a multislice HRTEM simulation with 20% added noise produced from the model in Figure 5(c) is given, and in Figure 5(e–g), detail **I** from the ABSF image in Figure 5(b), detail **II** from the structure model in Figure 5(c), and detail **III** from the multislice simulation in Figure 5(d) are presented consecutively. These three items, in plan view, suggest the shortest-term lowest-energy configuration and disposition of the Cs^+^ cations around the [Mo_6_Br_14_]^2–^ anions within a 14 Å diameter SWCNT.

**Figure 6:**
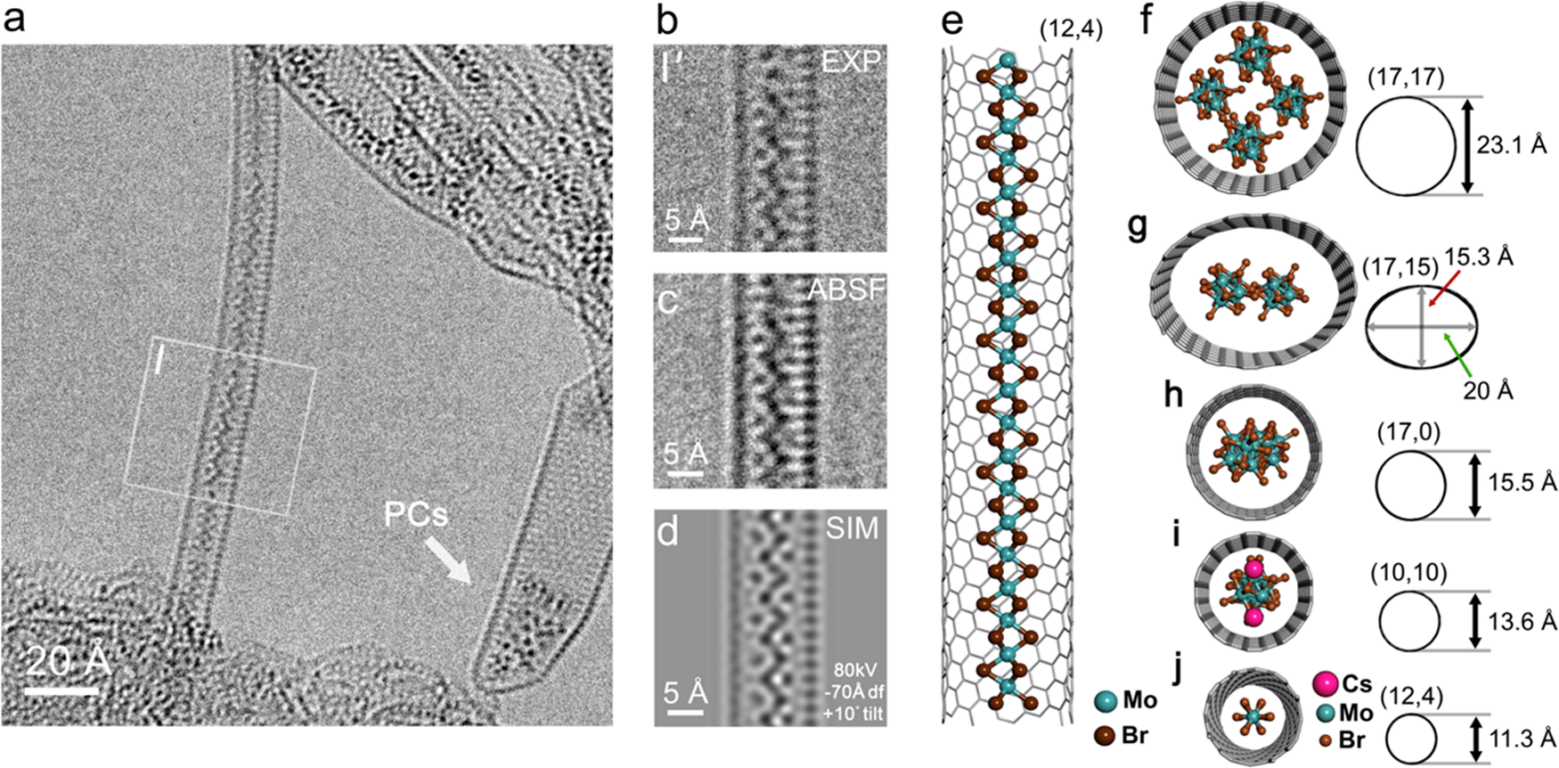
(a) HRTEM image of a chiral ∼11.3 Å diameter SWCNT encapsulating a continuous polymerized [Mo_2_Br_6_]_*x*_ chain (left). A wider funnel-shaped SWCNT containing a random group of packed clusters (PCs) is at right. (b, c) Detail and ABSF filtered version of the indicated region **I** in (a). (d) Multislice simulation obtained from the model in (e), which corresponds to a 10° tilted polymerized [Mo_2_Br_6_]_*x*_ chain in a chiral (12,4) SWCNT, showing that the nanotube wall carbons are resolved on one side only, a clear indication of chirality.^19,43^ (f) Cross-section of the model for Figure 1(h) with SWCNT dimensions indicated. (g) Cross-section of the elliptically distorted Figure 3(b) model with SWCNT dimensions indicated. (h) Cross-section of the narrower (17,0) SWCNT region in Figure 4(c), bottom model. (i) Cross-section of ∼13.6 Å diameter SWCNT in Figure 5(a), the only image in which the Cs^+^ ion packing is visible together with the [Mo_6_Br_14_]^2–^ ions. (j) Cross-section of the (12,4) SWCNT suggested for parts (a–e) of this Figure. Note that the SWCNT is now too narrow to accommodate Cs_2_Mo_6_Br_14_ and a polymerized [Mo_2_Br_6_]_*x*_ chain is obtained instead.

We were also able to identify nanowires formed inside SWCNTs with diameters too small to accommodate the [Mo_6_Br_14_]^2–^ anions and Cs^+^ cation pairs in the cross-section. In these cases, a complex process of chemical elimination seems to have occurred, giving rise to the formation of continuous [Mo_2_Br_6_]_*x*_ polymer chains in SWCNTs. In Figure 6(a), an HRTEM image of a 150 Å-long section of SWCNT continuously filled with polymerized [Mo_2_Br_6_]_*x*_ is shown. At the right, a group of randomly orientated [Mo_6_Br_14_]^2–^ anions in a funnel-shaped SWCNT is visible, similar to the packed [Mo_6_Br_14_]^2–^ anions in Figure 4(a,b). This nanowire, visible in enlarged experimental (EXP) and ABSF filtered detail in Figure 6(b,c), respectively, is found in a chiral (12,4) SWCNT with a diameter of 11.4 Å. This conformation SWCNT was chosen as wall carbon spacing can be seen on only one side of the SWCNT,^19,43^ and its precise diameter (calculated as 11.293 Å)^44^ is close to what we observed experimentally. A multislice simulation and corresponding model are reproduced in Figure 6(d,e), respectively. This was the smallest structure that we observed so far and was only found in the narrowest SWCNTs. In Figure 6(f–j), we depict the reverse evolutionary packing behavior of the [Mo_6_Br_14_]^2–^ cluster-based ions (Cs^+^ species are omitted from Figure 6(f–h) for clarity) as the SWCNTs narrow from ∼23 to ∼11 Å, as observed in Figures 1 and 3–6, respectively, each presented with a diagrammatic representation of the SWCNT dimensions including the observed elliptically distorted SWCNT in Figure 3 as shown in Figure 6(g).

**Figure 7:**
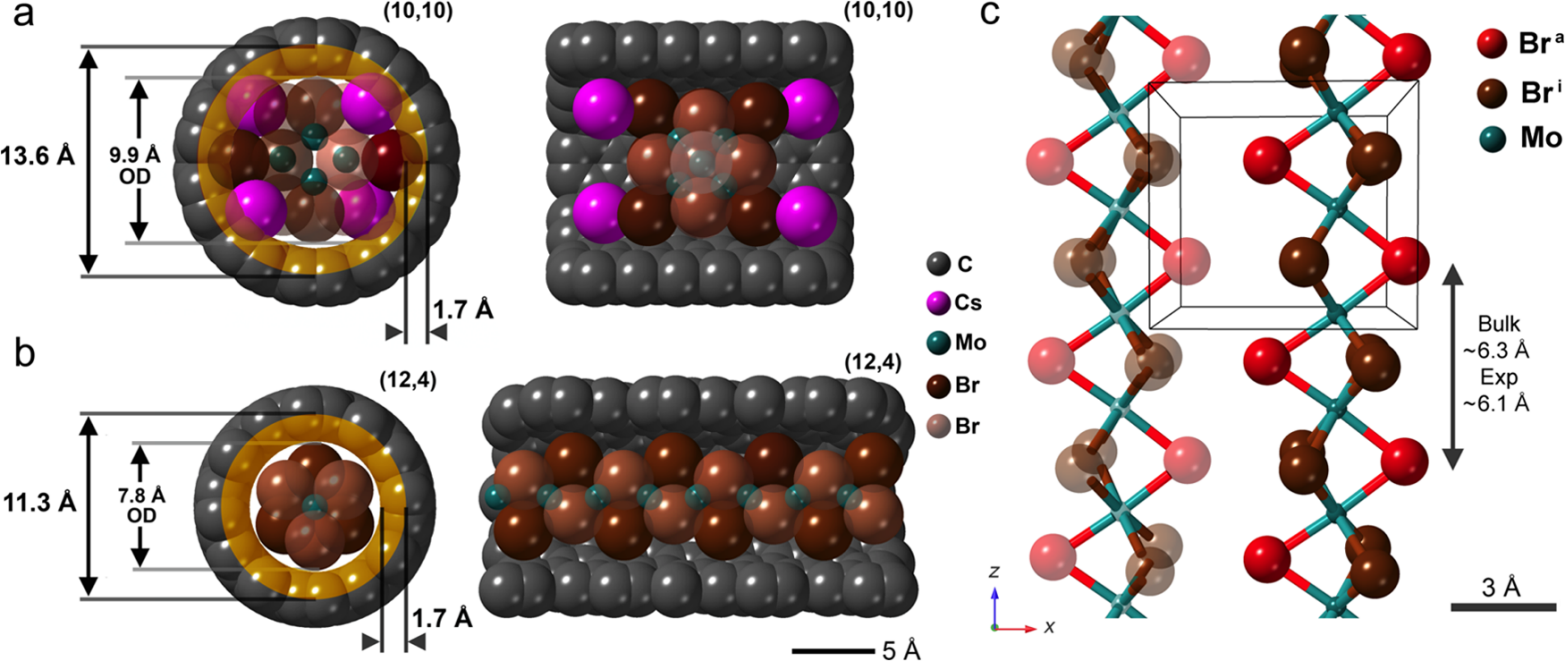
To scale space-filling models of Cs_2_Mo_6_Br_14_@(10,10) and [Mo_2_Br_6_]_*x*_@(12,4) SWCNTs. (a) End on (left) and side on a cutaway view (right) of a space-filling model of a Cs_2_Mo_6_Br_14_ anion in a (10,10) SWCNT with the tubule diameter, approximate outside diameter (OD) and inner 1.7 Å wide volume of the tubule indicated. The atomic arrangement (side on view) is as in Figure 5(f). (b) as for (a) but with the [Mo_2_Br_6_]_*x*_ polymer inserted into a narrower (12,4) SWCNT. Overall clearance is ∼0.1 Å if the radii chosen are standard Shannon ionic radii for C, Mo^3+^, and Br^–^. In all models, the foremost Br^–^ anions are faded such that the 3D atomic arrangements of Mo are easier to visualize. (c) Depiction of part of the orthorhombic 2D Ising model for MoBr_3_ with the RH 1D [Mo_2_Br_6_]_*x*_ chain indicated and an adjacent second LH [Mo_2_Br_6_]_*x*_ chain slightly faded. The SWCNT in Figure 6(a–e,j) and (b) can only accommodate a single such 1D [Mo_2_Br_6_]_*x*_ Ising-like chain by inspection.

The schematic SWCNT diameters depicted in Figure 6(i,j) indicate the nanoscale tubular structures for which we observe, on either side of a structural limit, either closely packed [Mo_6_Br_14_]^2–^ and Cs^+^ ions or continuous polymeric [Mo_2_Br_6_]_*x*_ although the nanotube diameters alone do not address the spatial relationships. The close correlation between the internal volume of a SWCNT and the stability of an atomic, molecular, or crystalline species^1−20^ is regulated by the tubule diameter in close association with the carbon van der Waals surface.^45^ However, as we see in Figure 3 and elsewhere,^10,45−47^ a molecular assembly or crystalline species that is asymmetric in cross-section can also elliptically distort the SWCNT cross-section. On the other hand, we see that in Figures 5 and 6, the SWCNTs are apparently undistorted, so the spatial relationships are mostly reduced to interactions between encapsulates, SWCNT diameter, and its surfaces. In space-filling models depicted in Figure 7(a,b), based on the directly observed composites in Figures 5(e,f) and 6(a–c), with atom sizes derived from standard Shannon values for Cs^+^, Mo^3+^, Br^–^, and neutral C (Table S1^48^), we see more closely the interactions between armchair (10,10), chiral (12,4), SWCNTs, Cs_2_Mo_6_Br_14_, and polymeric [Mo_2_Br_6_]_*x*_, respectively. It is clear from these models that once the carbon 1.7 Å van der Waals radii (yellow shaded region in Figure 7(a)) are taken into account, the (10,10) SWCNT can barely just accommodate the Cs_2_Mo_6_Br_14_ species and will probably incur some distortion in the latter. This feature is difficult to verify because of the low visibility of Br^–^ in the HRTEM images. In the case of the narrower (12,4) SWCNT, the accommodation of the [Mo_2_Br_6_]_*x*_ is almost perfect and, including the 1.7 Å van der Waals radii (yellow shaded region in Figure 7(b)), we see a ∼0.1 Å net clearance around the polymer chain. A further depiction of the proposed packing of Cs_2_Mo_6_Br_14_ as observed in the wider (10,10) SWCNTs is reproduced in Figure S1.

The observed [Mo_2_Br_6_]_*x*_ polymer is derived from the bulk molybdenum bromide structure, which, similar to TiI_3_ and related halides,^49^ consists of the crystal structure of parallel infinite columns of face-sharing [MoBr_6_] octahedra in either hexagonal or orthorhombic arrangements in two dimensions. In a further representation in Figure 7(c), we see a ball-and-stick representation of a 1D [Mo_2_Br_6_]_*x*_ chain in an orthorhombic *Pnmm* unit cell with the apical (Br^a^) and equatorial (Br^i^) bromine atoms indicated with an additional (faded) chain from the same structure indicated. There is a strong correlation between the experimentally observed Br^a^ – Br^a^ separation, which is ∼6.3 Å, while in corresponding chains in the orthorhombic bulk form, this distance is ∼6.1 Å (i.e., Figure 7(c)). In order for the [Mo_2_Br_6_]_*x*_ polymer to form from Cs_2_Mo_6_Br_14_, a collective elimination of “Cs_2_Mo_4_Br_8_” per Mo_2_Br_6_ monomer must occur with a net shift in the oxidation state from Mo^II^ (bulk form) to Mo^III^ (monomer) perhaps also accompanied by the formation of some Mo^0^ in order to maintain chemical balance. Changes in the oxidation state and/or stoichiometry are not uncommon in numerous SWCNT encapsulates formed by apparently neutral chemical processes. For example, reduced stoichiometry and oxidation state La^II^I_2_ formed by liquid phase insertion of La^III^I_3_ was reported,^50^ continuously variable stoichiometry in TbBr_*x*_ (with *x* varying from 2.8 to 2.0), linked to shifts in the SWCNT diameter^51^ and, more recently, encapsulated halide perovskite compositions of Cs_3_Pb^II^X_3_ (X = Br or I) were observed when the composition CsPb^II^X_3_ was introduced into nanotubes.^21,22^ These changes in expected stoichiometry can mostly be linked to adjustments in surface chemistry that occur due to spontaneous crystallization and confinement, although these phenomena do not account for observed chemical elimination, which must be counterbalanced with complementary shifts in stoichiometry elsewhere in any sample where this process is observed. Additionally, in order for each “Cs_2_Mo_4_Br_8_” eliminated per formula unit to occur, we anticipate that this species could in fact be separated into its known stable constituents, for example, CsBr, Mo_6_Br_12_, and Mo^0^, yielding the globally balanced process, with all of the additional species potentially forming either inside or outside other SWCNTs. Apart from Cs_2_[Mo_6_^II^Br_14_] and polymerized [Mo_2_^III^Br_6_]_*x*_, we have not so far positively identified any of the additional anticipated species, but their proposed formation is the subject of ongoing investigations.



In MoBr_3_ (i.e., “Mo_2_^III^Br_6_”), in its orthorhombic form (Figure 7(c)), along with analogous materials such as TiI_3_, which forms both *Pnmm* and hexagonal *P*6_3_/*mcm* forms, based on alternative stackings of 1D [Mo_2_Br_6_]_*x*_ or [Ti_2_I_6_]_*x*_, chains have been studied as possible 2D Ising lattice systems,^49,52,53^ which are of interest in statistical mechanics and in forming ferromagnetic arrays. Isolating a single [Mo_2_Br_6_]_*x*_ chain, as we observe in Figures 6 and 7, formed in this case by nanoconfinement and elimination from Cs_2_Mo_6_Br_14_ but which are now structurally isolated with respect to bulk MoBr_3_, represents the formation of a possible candidate for a true 1D Ising-like crystal.^52,53^ 2D Ising-like MoBr_3_ is proposed to consist either of orthogonal or hexagonal arrays effectively reduced to 1D by SWCNT confinement. It would be easier to form such materials directly from MoBr_3_, although crystallization in the right form is not always guaranteed, and our indirect method provides a route to forming such crystals in high quality.

It is informative to compare our results with similar results recorded for the anion [Mo_6_I_14_]^2–^, which was also imaged inside SWCNTs but was inserted using a different method.^20^ In this previous study, [Mo_6_I_14_]^2–^ was formed by reacting Mo(CO)_6_ inserted into SWCNTs with iodine to form the [Mo_6_I_14_]^2–^ cluster anion (but without Cs^+^ counterions) and then subsequently reacted with H_2_S to form [MoS_s_]_*n*_ nanoribbons. An additional noteworthy feature of this investigation is that the range of SWCNTs, in terms of diameter, was kept very narrow, with a mean tube diameter (*d*_t_) of 1.4 nm. From the standpoint of our study, the most interesting aspect of this work was the formation of the [Mo_6_I_14_]^2–^ intermediate, which is isostructural with [Mo_6_Br_14_]^2–^ and which showed very similar imaging properties which were also correlated with a tilt tableau similar to Figure 2. Although some polymerization of the [Mo_6_I_14_]^2–^ monomer was reported, the observed polymeric chain consisted of a near-linear chain of Mo_6_I_14_ units, which are approximately double the outside diameter (i.e., 7.8 Å) of our Ising-like [Mo_2_Br_6_]_*x*_ chain as shown in Figure 7(b). These results are remarkable in the sense that, combined with the current study, we see in more detail the greater flexibility of chemistry that can be performed due to steric confinement in SWCNTs.

### 2.2 Raman Spectra

**Figure 8:**
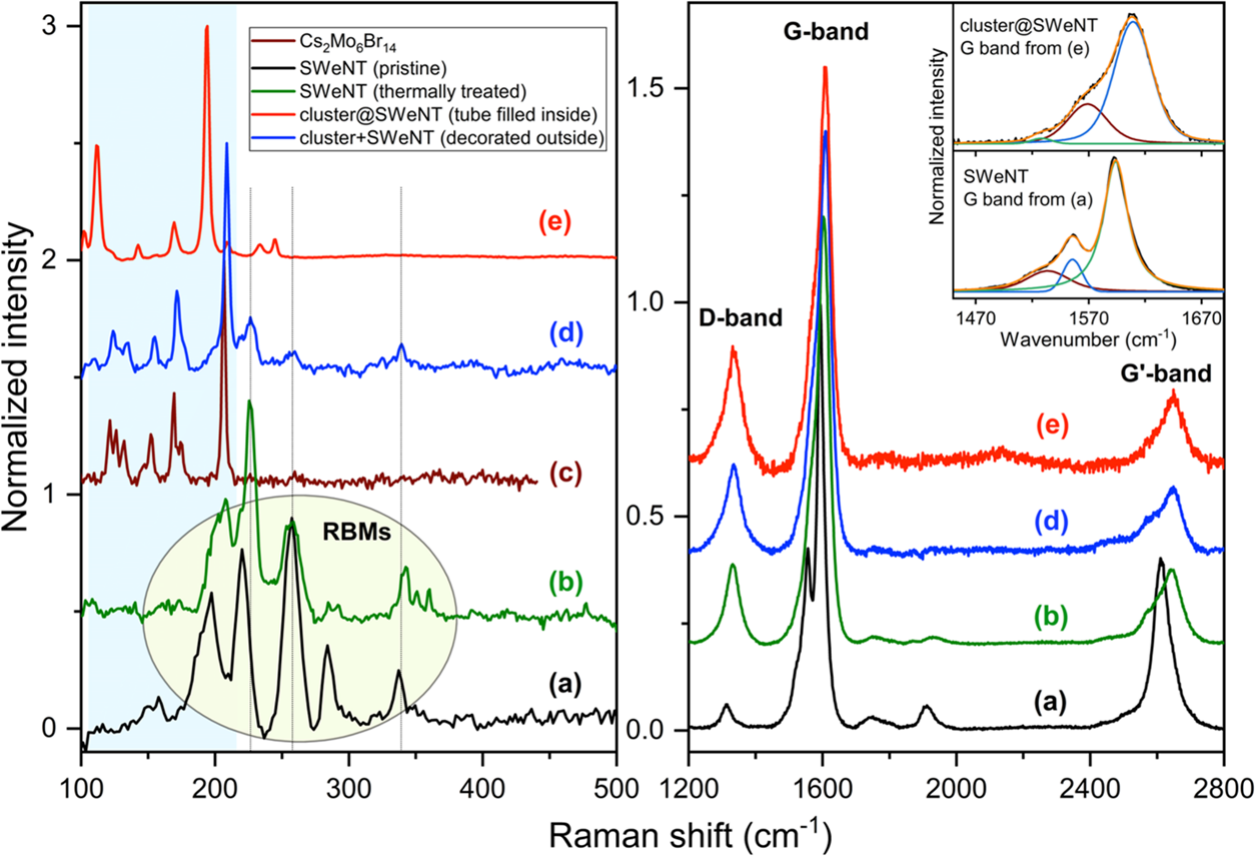
Raman spectra of cluster and SWeNT composite for λ_exc_ = 632.8 nm. (a) pristine SWeNT nanotubes; (b) annealed empty SWeNT nanotubes; (c) pristine Cs_2_Mo_6_Br_14_ crystallites; (d) Cs_2_Mo_6_Br_14_ cluster/SWeNTs aggregates with cluster moieties outside the SWeNTs walls; and (e) cluster@SWeNTs composite with cluster moieties inside the nanotubes. Inset: fits of the G bands with three Voigt components for spectra (a, e). See also Figure S2.

Figure 8(a–e) displays a series of Raman spectra obtained using a LabRam spectrometer from various SWCNT samples: pristine tubes, hybrid samples (composites), and Cs_2_Mo_6_Br_14_ crystallites, all excited at 1.959 eV (633 nm). These spectra are separated into two parts, with the radial breathing mode region on the left (i.e., Raman shifts 100 to 500 cm^–1^) and the D, G^–^/G+, and G′ bands on the right (i.e., Raman shifts 1200 to 1800 cm^–1^). These Raman spectra provide valuable information about the different samples and their structural characteristics, allowing us to distinguish between pristine SWCNTs, empty and filled SWCNTs with clusters, and the Raman signatures associated with the clusters themselves.

#### 2.2.1 Low-Frequency Raman Lines of Pristine SWCNTs

In Figure 8(a), we observe that the RBM frequencies of the pristine SWCNTs appear at 158, 197, 220, 258, 284, and 337 cm^–1^. Notably, the RBM lines in the pristine and empty annealed CNT samples (Figure 8(a,b)) occur at nearly the same positions, suggesting that the heating process does not significantly affect their diameter or chirality. Using the empirical relationship between the RBM frequency ω_RBM_ (in cm^–1^) and tube diameter *d*, expressed as *d*(nm) = 234/ω_RBM_, we can estimate that these peaks correspond to SWCNTs with diameters ranging between 0.69 and 1.48 nm, as shown in Table 1 (see also Figure S2). The most intense RBM line gives a diameter of *d* = 0.91 nm, confirming that the commercial nanotubes selected for synthesizing our composites belong to the dominant crystalline structural forms probed with the laser excitation energy of 1.96 eV in the analyzed samples. Moreover, the larger diameters present in the distribution can easily accommodate the cluster molecules, which have a size of 0.89 nm.

**Table 1:** Estimated SWCNT Diameters (nm) from Their RBM and TO Mode Frequencies (cm^–1^)

ω (RBM)	*d*	ω (TO)	*d*
158	1.48	1524	0.69
177	1.32	1528	0.71
187	1.25	1534	0.76
197	1.19	1556	1.03
199	1.18	1559	1.09
220	1.06	1569	1.45
227	1.03		
254	0.92		
258	0.91		
266	0.88		
284	0.82		
285	0.82		
337	0.69		

#### 2.2.2 High-Frequency Raman Lines of the Pristine SWCNTs

The tangential G band of pristine SWCNTs exhibits three components at 1534, 1556, and 1594 cm^–1^. In this paper, we assign these components based on the work of Telg et al.,^54^ who studied the chiral index dependence in semiconducting SWCNTs. They demonstrated that the highest frequency mode (G^+^) is nearly independent of the tube diameter, *d*, and close to the E_g_ mode in graphite (1582 cm^–1^). This mode is a longitudinal optical (LO) mode that is minimally affected by tube curvature. In contrast, the transverse optical (TO) mode (G^–^) strongly depends on curvature, and its Raman frequency varies with *d*, following the empirical relationship ω_G^–^_(*d*) = *a*_0_ + *a*_1_/*d*^2^ with *a*_0_ = 1582 cm^–1^ and where *a*_1_ = −27.5 nm^2^/cm is a chirality dependent parameter.^55^

Thus, it is reasonable to assign all modes above 1582 cm^–1^ to G^+^(LO) modes and those below to G^–^(TO) modes. In our case, the highest frequency band at 1594 cm^–1^ can be attributed to a G^+^(LO) mode, while the other bands at 1534 and 1556 cm^–1^ correspond to G^–^(TO) modes, originating from tubes with different diameters (see Table 1).

#### 2.2.3 Cluster Raman Lines

The Raman lines of cluster powder recorded in our experiments appear at frequencies 65, 71, 121, 126, 132, 152, 170, 174, and 207 cm^–1^ (Figure 8(c)), which are well reproduced with DFT calculations on relaxed in vacuo structures of the [Mo_6_Br_14_]^2–^ ion. No lines were found at higher frequencies. The spectra were recorded here at room temperature, and the Raman lines and intensities agree with earlier results obtained at 80 K by Garreau.^58^ In both studies, the most intense Raman line occurs at 207 cm^–1^. However, this author was able to record weak Raman lines with A_1g_ symmetry in the range 322–328 cm^–1^ that have not been observed in the present work.

#### 2.2.4 Low-Frequency Lines of Nonencapsulating SWCNT Composites

The cluster lines in the composite analyzed in Figure 8(d) do not shift with respect to those of the cluster powder investigated. This suggests that there is a weak interaction between the cluster and the CNT. In this case, the cluster is possibly lying outside the CNT, between bundles or individual CNTs, which is supported by the presence of remnant relatively intense RBM lines of the SWeNT SWCNTs at 226, 257, and 337 cm^–1^.

#### 2.2.5 Raman Lines of the Encapsulating “SWeNT” SWCNT Composites

Upon analyzing the spectra in Figure 8(e) obtained from another composite sample, we observe the simultaneous presence of cluster lines and the D-G-G′ bands, similar to the previous composite case shown in Figure 8(d). However, there is no indication of RBM signals originating from the SWCNTs, suggesting that some RBM modes are suppressed (cf. Figure 8(a,b)), which points to a high filling rate in “SWeNT” SWCNTs. Notably, the most prominent line in the cluster spectrum, attributed to the internal breathing motions of the inner Br ligands (Br^i^), exhibits a downshift in frequency toward lower values (193 cm^–1^) compared to the isolated cluster spectrum (207 cm^–1^). This observation suggests a stronger interaction occurring between the clusters and “SWeNT” SWCNTs. Furthermore, the absence of RBM modes from SWCNTs in some samples, as illustrated in Figure 8(e), suggests that the Raman spectra likely represent clusters (or, alternatively, the [Mo_2_Br_6_]_*x*_ polymer) encapsulated within SWCNTs. The encapsulation hinders the RBM vibrations due to the increase in radial stiffness of the tubes. This finding provides additional evidence for the tight confinement of clusters within the SWCNTs, leading to significant modifications in their vibrational behavior and strengthening the interaction between the clusters and the SWCNTs. It is seen that the tangential G^+^ band (LO mode) experiences a significant upshift from 1592 cm^–1^ in pristine tubes to 1610 cm^–1^ upon cluster intercalation, while the G^–^ band gradually merges into the main band, resulting in an unresolved line at around 1571 cm^–1^. Empty tubes that have undergone only the heating process without intercalation, as manifested by the absence of cluster lines in their spectra, present G^–^ and G^+^ bands shifted from 1556 to 1563 cm^–1^ and from 1592 to 1604 cm^–1^, respectively. Therefore, there is a significant influence of the insertion of Cs_2_Mo_6_Br_14_ on the vibrational and electronic properties of SWCNTs after intercalation or insertion. The C–C bonds of the SWCNTs apparently stiffen consistent with a slight shortening of the C–C bond lengths.

The G′ (2D) mode is a fingerprint Raman feature of aromatic sp^2^ carbon phases and is related to a second-order scattering from the aromatic ring breathing vibrations belonging to the edge of the Brillouin zone (BZ). Its intensity, comparable to that of the G mode, is a result of the double resonance between the incoming and the scattered photons with electron transitions in the vicinity of the Dirac cone. Any kind of structural defects leading to relaxation of the excited electronic states involved in the double resonance result in the widening of the G′ band and a shift of its spectral maximum due to the involvement of vibrations from inside of the BZ. It has been demonstrated^56,57^ that the spectral shape of the G′ mode is very sensitive to nanoscale deformations of the CNTs or the graphene sheet, like bending, twisting, or bubble formation. Notably, bubbles are observed by TEM in our CNTs, as shown in Figure 4. Bending and elliptical deformations of the CNTs cross-section are present as well, as evident from Figure 3 and also Figure 6.

#### 2.2.6 Additional Raman and Luminescence Spectra

A second set of Raman Spectra recorded from Cs_2_Mo_6_Br_14_ treated “SWeNT” SWCNTs is reproduced in Figure S2, which were obtained with a T64000 spectrometer (Experimental Section) in triple subtractive monochromator configuration, with an excitation set at 1.916 eV (647.1 nm), for both pristine single-walled carbon nanotubes (SWeNT) and composite tubes infiltrated with the cluster material. With this configuration, it was possible to record frequencies below 100 cm^–1^ and observe a strong photoluminescence (PL) background in the Raman spectra of the filled samples, as shown in Figure S2(a). These spectra also exhibited cluster Raman lines alongside weakly intensified G bands. Based on HRTEM evidence, which indicated that clusters can be encapsulated intact with strong packing interactions within nanotubes of approximately 2 nm diameter (Figure 1), it is reasonable to attribute the luminescence to broad-filled nanotubes. In contrast, narrow nanotubes filled with polymerized clusters that transform into continuous [Mo_2_Br_6_]_*x*_ chains (i.e., as shown in Figure 6) do not exhibit luminescence, thus explaining the absence of luminescence in those samples.

**Figure 9:**
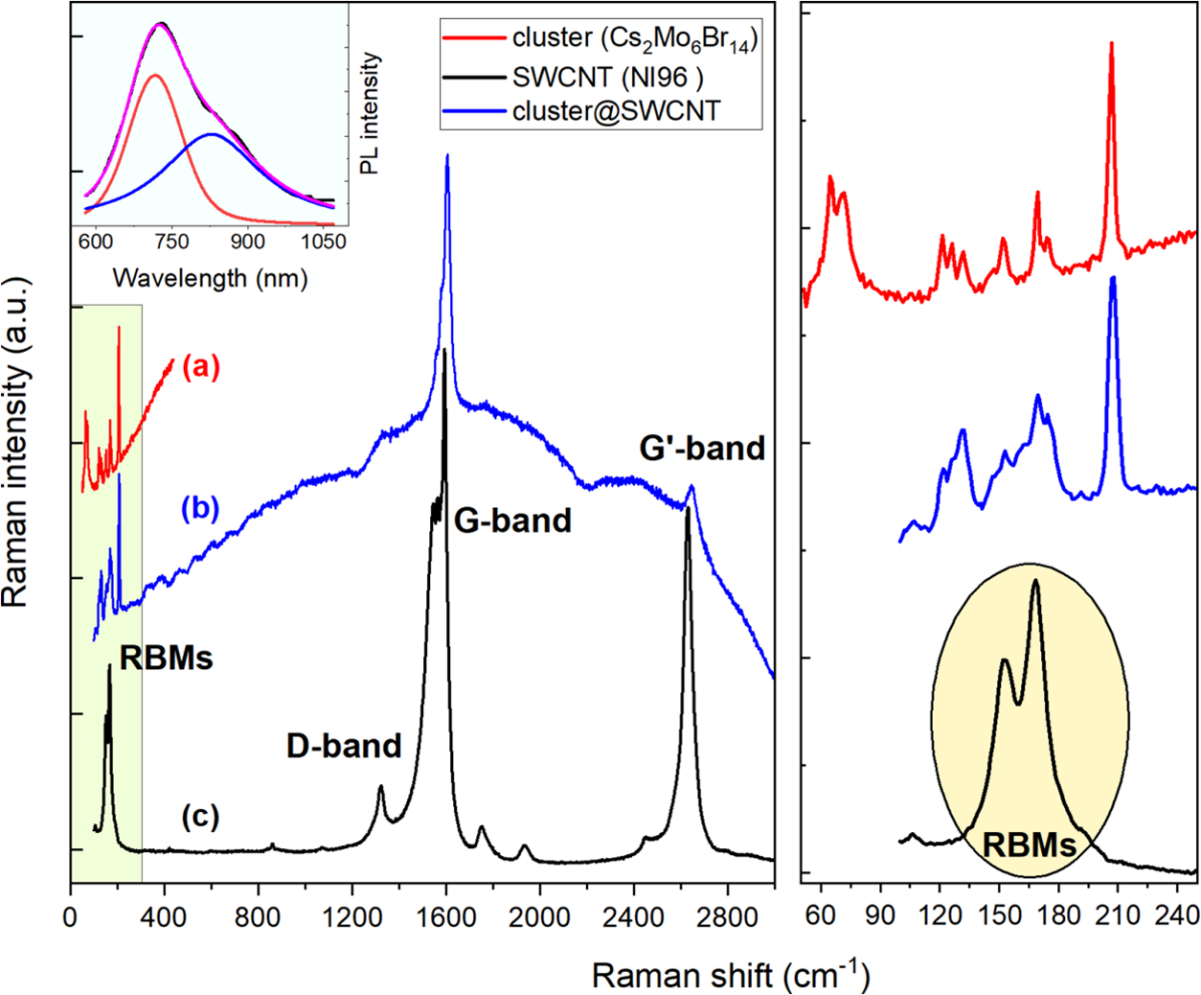
Raman spectra of cluster and cluster@NI96 SWCNT composite for λ_exc_ = 632.8 nm. Left panel: (a) raw spectra of pristine cluster Cs_2_Mo_6_Br_14_ crystallites; (b) cluster@NI96 SWCNT composite; (c) pristine NI96 SWCNT. Inset left: photoluminescence spectrum of the pristine cluster at room temperature and excitation at 510 nm. Right panel: close-up of the low-frequency regions.

In the top panel of Figure S2(a), we compare the Raman spectra of pure Cs_2_Mo_6_Br_14_ and that of cluster@SWeNT samples recorded during the experiment. The baseline of this background in the composite sample follows the fluorescence spectrum of clusters, ranging from 647.1 nm (corresponding to a Raman shift, ω = 0 cm^–1^) to 743.3 nm (ω = 2000 cm^–1^), as depicted in the top left inset of Figure 9 for *T* = 295 K. As a result, the Raman spectrum of the cluster@SWeNT material displays sharp Raman contributions from the cluster, superimposed on the fluorescence spectrum of the cluster itself. Notably, the RBM bands between 170 and 300 cm^–1^ completely vanish, while other lines occur only around 327 and 354 cm^–1^, corresponding to SWCNT diameters of about 0.7 nm. Despite the strong PL background, the G Raman bands of the SWCNTs are still visible, albeit with a weak intensity and lacking distinct features. In Figure S2(b), it is also evident that the relative intensity of the D band (1352 cm^–1^), indicative of morphological defects, increases after treatment, implying the creation of more defect states during the infiltration process, and reveals a significant upshift compared to pristine SWCNTs (1310 cm^–1^).

Figure 9 shows the Raman spectra for both pristine single-walled carbon nanotubes (SWCNTs) from NanoIntegris (NI96, 96%) and a composite of these tubes infiltrated with the cluster material with Raman excitation set at 1.959 eV (632.8 nm). For comparison, we also present the Raman spectrum of the cluster compound, recorded using the T64000 spectrometer (see the Experimental Section) in a triple subtractive monochromator configuration. This setup enabled the detection of cluster-related frequencies below 100 cm^–1^.

In both the treated NI96 SWCNTs and the cluster material, we observed also a pronounced PL background in their Raman spectra. The spectrum of the composite sample displayed Raman peaks corresponding to the cluster in addition to the characteristic G bands of the nanotubes, which were notably reduced in intensity compared to the pristine NI96 spectrum. The composite also showed two radial RBM at 153 and 168 cm^–1^, corresponding to nanotubes with diameters of 1.48 and 1.35 nm, respectively—much broader than those seen in the SWeNT nanotubes investigated in Figure 8. Based on HRTEM evidence, which indicated that clusters can be encapsulated intact with strong packing interactions within nanotubes of approximately 2 nm diameter (Figure 1), it is reasonable to attribute the luminescence signal to broad-filled NI96 nanotubes. In contrast, narrow SWeNT nanotubes filled with polymerized clusters that transform into continuous [Mo_2_Br_6_]_*x*_ chains (i.e., as shown in Figure 6) do not exhibit luminescence, thus explaining the absence of luminescence in those samples (see Figure 8).

In the composite sample, the strong fluorescence background closely mirrors the PL spectrum of the clusters, as shown in the inset of Figure 9 for *T* = 295 K. Consequently, the Raman spectrum of the cluster@NI96 material displays sharp Raman peaks corresponding to the cluster, superimposed on its PL spectrum. Notably, the RBM bands observed in the pristine NI96 spectrum disappear completely in the composite (right panel of Figure 9). Despite the overwhelming PL background, the G/G′ bands of the treated NI96 SWCNTs remain visible, albeit with reduced intensity and less distinct features.

It is worth noting that the intrinsic fluorescence of SWCNTs cannot be detected in the Raman spectra produced by our samples. The spectral range of SWCNT PL, when excited at 647.1 nm, should appear at higher wavelengths, specifically between 900 and 1600 nm. Additionally, the aggregation of SWCNTs into bundles is known to quench intrinsic nanotube fluorescence, which possibly explains the absence of SWCNTP PL in our Raman measurements.

### 2.3 Structural and Vibrational Calculations for the [Mo_6_Br_14_]^2–^ Ion and the [Mo_2_Br_6_]_*x*_ Chain

The free cluster ion belongs to the *O_h_* symmetry, similar to the observed SWCNT encapsulated [W_6_O_19_]^2–^ Lindqvist ions^18,19^ and isostructural [Mo_6_I_14_]^2–^ ions, also observed in SWCNTs.^20^ The Raman modes of this ion are represented by
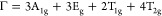


The structure of the [Mo_6_Br_14_]^2–^ ion was relaxed with DFT, giving Mo–Br^a^, Mo–Br^i^, and Mo–Mo distances of 0.259, 0.258, and 0.263 nm, respectively. The Raman lines obtained from the cluster powder in our experiments (Figure 8(c)) are in good agreement with the results of DFT calculations performed on relaxed in vacuo structures of the [Mo_6_Br_14_]^2–^ ion. The corresponding vibrational frequencies, along with their assignments, are listed in Table 2. According to DFT predictions, the intense line observed at 207 cm^–1^ corresponds to the breathing mode of the inner Br^i^ atoms, calculated at 202 cm^–1^. The breathing mode of the outer Br^a^ atoms is calculated at 151 cm^–1^.

**Table 2:** Theoretical and Experimental Raman Active Frequencies (in cm^–1^) of the Molybdenum Bromide Compounds Investigated in This Study^a^

	theory			experiment		
free [Mo_6_Br_14_]^2–^ cluster-based unit		[Mo_2_Br_6_]_*x*_ chain		bulk Cs_2_Mo_6_Br_14_	cluster outside tube	MoBr phase inside tube
mode symmetry	frequency	mode symmetry	frequency	frequency	frequency	frequency
T_2g_ Mo–Br^a^ scissoring	40.6		80.3			
			88.1			
		A_2_	96.4			
			98.9			
			100.9			
T_2g_ Mo cage deformation	117.2	E Mo–Br^i^ bend	102.4	121	109	
E_g_ Mo–Mo stretching	126.5	E Mo–Br^i^ bend	117.7	125	124	114
			120.2	134	134	
			152.9	152	155	144
			159.2			
A_1g_ Br^a^ BM^a^	164.3		163.1			
T_1g_ Br_a_ bend	171.6					
E_g_ Mo–Br^a^ stretching	178.4		170.1	169, sh.^b^175	171, sh.176	169
		A_1_ Mo–Br^a^ stretching	193.1		sh.196	
		E Mo–Mo bending	197.9			194
T_2g_ Mo–Br^i^ stretching	196.4		250.9			
A_1g_ Br^i^ BM	214.3			207	209	213
						233
T_1g_ Mo–Br_i_ stretching	251.4					245
E_g_ Mo–Mo stretching	264.5		267.2		259	
A_1g_ Mo BM	317.1	A_1_ Mo–Mo stretching	323.2			328 weak
T_2g_ Mo cage deformation	349.2				339	

a BM: breathing mode.

b sh.: shoulder.

In our DFT model, the elongations of the Mo–Mo framework are calculated as 257 and 339 cm^–1^, with a breathing mode appearing at 356 cm^–1^. However, in the experimental data, only a very weak Raman signal is detected midst the noise within this frequency range. Garreau successfully recorded this mode with a weak Raman intensity, and his DFT calculation of the isolated [Mo_6_Br_14_]^2–^ ion aligns well with our findings.^58^

In Section 2.1, we have identified the most probable 1D MoBr structure in very narrow SWCNTs as the [Mo_2_Br_6_]_*x*_ chain. This isolated specific trigonal chain structure (i.e., as depicted in Figure 7(b)) belongs to the space group *P*3*m*1 (#156) as opposed to the bulk 2D *Pnnm* form (i.e., Figure 7(b), #59). For our investigations, DFT calculations were initiated from this [Mo_2_Br_6_]_*x*_ chain structure, using a translational *c* parameter of 6.06 Å, Mo–Mo distances of 2.92 and 3.14 Å, and a Mo–Br bond of 2.57 Å. These distances are comparable to those found in the MoBr chains of the MoBr_3_ 3D crystal with *c* = 6.08 and 2.59 Å for Mo–Br bonds. To ensure the structural stability of the standalone chain, we further optimized the parameters, resulting in a revised configuration with *c* = 5.68 Å, Mo–Mo distances of 2.31 and 3.37 Å, and a Mo–Br distance of 2.66 Å. The most notable change is the reduction of *c*, which tends toward a smaller value than that observed in HRTEM (∼6.3 Å).

From this structure, the Wyckoff positions of the two Mo atoms and the six Br atoms in the unit cell are denoted as 1a and 3d, respectively. Performing factor group analysis, we obtain the following mechanical and vibrational representations
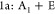

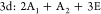

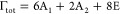

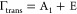

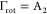

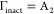

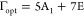


Active Raman modes in the system can be classified into either A_1_ or E representations. Table 2 provides a summary of the experimental and theoretical frequencies for the Mo_2_Br_6_ chain, along with their respective assignments. Based on DFT calculations of the relaxed chain structure, the most likely Raman lines associated with the [Mo_2_Br_6_]_*x*_ chains encapsulated in narrow SWCNTs are observed at 114, 144, 169, 194, 245, and 328 cm^–1^. The corresponding DFT-calculated frequencies are approximately 117.7, 152.9, 170.1, 193.1, 197.9, 250.9, and 323.2 cm^–1^, respectively.

## 3 Conclusions

In this study, we have directly observed the complex packing of encapsulated structures of the phosphorescent Cs_2_Mo_6_Br_14_ cluster-based halide in single-walled carbon nanotubes (SWCNTs) as a function of their variable diameter from ∼23 Å down to ∼11 Å. Imaging by HRTEM revealed in exceptional detail how this packing was reduced in cross-section as the SWCNT diameter became smaller, causing, in one case, elliptical distortion of the tubules and random packing in another but gradually tending toward linear packing as the narrowing nanotube diameter (*d*_t_) and van der Waals surface imposed a structural limit on Cs_2_Mo_6_Br_14_ accommodation. Below this limit, the Cs_2_Mo_6_Br_14_ composition is eliminated in a complex fashion and SWCNT confinement results in spontaneous polymerization forming chains of neutral [Mo_2_Br_6_]_*x*_ in tubules with *d*_t_ < ∼11 Å. This inorganic polymer can additionally be related to a 1D Ising-like crystal as distinct from its orthorhombic bulk counterpart, which has been investigated as a 2D Ising model lattice.^49,52,53^

Due to what we believe to be the exceptional mobility of the Cs^+^ cations under the influence of the electron beam, we were rarely able to directly image these counterions except when the SWCNT diameter approached the Cs_2_Mo_6_Br_14_ accommodation limit of around 14 Å. Only at this side of the structural limit were we fleetingly able to observe the packing of Cs^+^ around [Mo_6_Br_14_]^2–^. On the other side of the volume structural boundary for the formation of polymeric [Mo_2_Br_6_]_*x*_, we can assume that this cation is eliminated either in tandem with other fragments with a net composition of “Cs_2_Mo_4_Br_8_” per formula unit, which must happen in order for the polymer to form. We anticipate that this species could in fact be separated into stable constituents such as CsBr, Mo_6_Br_12_, and Mo^0^, causing net fluctuations in stoichiometry inside and outside the SWCNTs. We will attempt to investigate these compositional fluctuations caused by confined crystal growth behavior, which we observe here but which have also been reported for other materials.^21,22,50,51^

To complement our HRTEM data, we conducted Raman scattering experiments on a diverse range of SWCNT samples, which were either mixed with or infiltrated by clusters. Through a detailed examination of the Raman shifts of the cluster-based units [Mo_6_Br_14_]^2–^ and composites for each configuration, we discovered that encapsulation significantly altered the intrinsic cluster-based unit vibrations as well as the radial breathing mode (RBM) and tangential G band frequencies of the SWCNTs. The encapsulation of clusters nearly suppressed the RBM mode and caused the G band frequencies to shift toward higher values, indicating a stiffening of the composite structures. These studies were further supported by DFT analysis of the vibrational characteristics of the *O*_*h*_ symmetry [Mo_6_Br_14_]^2–^ anions, which could be used to identify analogous modes measured for “bulk” anions outside the SWCNTs, cluster moieties located outside the nanotube walls, and clusters encapsulated within SWCNTs or anions within them. DFT calculations reveal that some modes could also possibly be attributed to the spontaneously formed [Mo_2_Br_6_]_*x*_ polymer, which is determined to have markedly different bending and stretching modes (Table 2). These Raman measurements, therefore, offered additional evidence and insights into the presence and spatial distribution of clusters in the SWCNT samples. These results are consistent with findings from previous studies on encapsulated crystals or molecules, which were investigated using Raman spectroscopy and demonstrate that such encapsulated species induce stress on the carbon walls and strain on the confined species. Our observations during [Mo_6_Br_14_]^2–^ encapsulation align with this behavior. Additionally, the trends in structural and vibrational changes, as revealed by preliminary density functional theory calculations, further support and validate our observations.

In summary, our study provides crucial insights into the encapsulation of Cs_2_Mo_6_Br_14_ within SWCNTs, shedding light on the effects of confinement on cluster structural and vibrational properties. The combination of HRTEM and Raman spectroscopy techniques proved to be highly valuable in unraveling the encapsulation behavior and understanding the intricate interactions between clusters and SWCNTs. The confinement induced transmutation of Cs_2_[Mo_6_^II^Br_14_] and adjusted stoichiometries of materials such as CsPb^II^X_3_ to Cs_2_Pb^II^X_5_,^21,22^ La^III^I_3_ to La^II^I_2_,^50^ and Tb^III^Br_3_ to TbBr_*x*_^51^ are of fundamental interest because of the likely effect on their electronic, magnetic, or optical characteristics and all are worthy of extended detailed investigations. This research significantly contributes to the advancement of nanomaterials science and opens new avenues for the design and synthesis of novel composite structures with molecular scale-tailored properties.

## 4 Experimental Section

### 4.1 Preparation of Cs_2_Mo_6_Br_14_ and Introduction into SWCNTs

The Cs_2_Mo_6_Br_14_ halide was synthesized as reported in ref (37) and was encapsulated in various types of SWCNTs. The experiment focused on two types of SWCNTs: (i) NanoIntegris PureTubes, with a 96% purity level (NI96), fabricated using an arc discharge method, with a diameter range of 1.4–1.7 nm and a length of 300 nm to 4 μm, and (ii) SouthWest NanoTechnologies SWCNTs, with a 90% purity level (SWeNT), created by the CoMoCAT process, with a diameter range of 0.7–1.1 nm (with a mean *d*_mean_ of 0.83 nm) and with a dominant (7,6) chirality. The cluster-nanotube composites were fabricated by using infiltration protocols described previously.^45^ Prior to the filling, the SWCNTs were washed, dispersed in *N*-methyl-2-pyrrolidone (NMP), dried, and then preheated to 1173 K (900 °C) to remove the solvent and open the tubes. The solid powder of clusters and SWCNT were mixed under a glovebox and then sealed in silica quartz ampules under vacuum. The tubes were then heated in a furnace for 6 h to 1223 K (950 °C).

### 4.2 Electron Microscopy Imaging and Simulation

HRTEM experiments were carried out with a JEM-ARM 200F microscope operating at 80 kV equipped with a CEOS aberration corrector and a Gatan SC1000 ORIUS camera with a 4008 × 2672 pixel charge-coupled device (CCD) detector. Electron beam densities were adjusted to between 0.8 and 1.88 pA/cm^2^ to minimize damage to the Cs_2_Mo_6_X_14_ clusters, and images were acquired at magnifications between 600,000× and 1.2 M×. Multislice image simulations were performed using the program SimulaTEM^41^ using parameters representative of the JEM-ARM 200F microscope (e.g., kV = 80 kV, *C*_s_ = 0.001 mm). Simulation models were assembled with the commercial program Crystalmaker from representative anion and cation fragments produced from the published bulk Cs_2_Mo_6_Br_14_ crystal structure^37^ and DFT models together with nanotubes generated with the software TubeGen Online version 3.4.^59^

### 4.3 Raman Spectroscopy

Micro-Raman experiments were performed with Horiba Jobin-Yvon T64000 and LabRam spectrometers in triple subtractive and single monochromator modes with a diffraction grating of 1800 and 600 grooves/mm, respectively. Laser excitations λ_exc_ = 647.1 and 632.8 nm with respective energies at 1.92 and 1.96 eV were provided by Kr^+^-ion and helium–neon lasers, respectively. Spectra were collected at room temperature in the range between 30 and 3000 cm^–1^, which covers the vibrational frequencies of both the SWCNTs and the Cs_2_Mo_6_Br_14_ filling material. The diameter of the laser spot on the sample surface was about 2 μm for the fully focused laser beam with 50× objective magnification. The cluster@SWCNT samples, with cluster moieties located inside the nanotubes, were found to be homogeneous under an optical microscope without extraneous microcrystalline residues of the cluster phase. Raman instruments were calibrated against the Stokes Raman signal at 520.5 cm^–1^ on a pure Si(100) wafer surface. The spectral resolution was lower than or equal to 2 cm^–1^. In each experiment, several spectra were recorded from different spots in the samples.

### 4.4 DFT Calculations and Technical Details

DFT calculations on the isolated Mo_6_Br_14_^2–^ ion in vacuo were performed with Quantum Espresso (QE).^60^ The QE calculations were achieved within the “pure” semilocal Perdew–Burke–Ernzerhof (PBE) approximation with a plane-wave (PW) basis set. Norm-conserving optimized Vanderbilt (ONCV) pseudopotentials^61,62^ with a kinetic-energy cutoff of 70 Ry (952 eV) were chosen for all atoms. The periodic boundary conditions were imposed on a 20 Å × 20 Å × 20 Å superlattice to consider the isolated ion without any intercluster interaction, and consequently, a 1 × 1 × 1 *k*–point mesh was used to represent the basis PW functions. The structural and vibrational properties of the periodic [Mo_2_Br_6_]_*x*_ chain were also modeled by QE software with the same exchange-and-correlation functional, cutoff energy, and pseudopotentials as those used for the Mo_6_Br_14_^2–^ ion. The 1D nature of the systems was accounted for by imposing periodic boundary conditions on a 20 Å × 20 Å square superlattice arranged perpendicular to the chains. The translation period *c* along the chains was of the order of 6 Å, as suggested by HRTEM, and was allowed to relax during the structure optimization. Correspondingly, a 1 × 1 × 16 *k*–point Monkhorst–Pack mesh was chosen, which is typical for molecular 1D crystals similar to the [Mo_2_Br_6_]_*x*_ chains under consideration.

## Supporting Information Available

Shannon ionic or atomic radii employed in the structure models; orientation scheme for Cs_2_Mo_6_Br_14_ outside or inside SWCNTs; Raman spectra for *l*_exc_ = 647.1 nm; and additional references (PDF)
Sequence showing vibration in position of the array of [Mo_6_Br_14_]^2–^ anions featured in Figure 1(e) (Movie S1) (AVI)
Sequence showing motion of [Mo_6_Br_14_]^2–^ anions packed into an outlier double-walled carbon nanotube (DWCNT) (Movie S2) (AVI)
Sequence showing the motion of a “zigzag” array of [Mo_6_Br_14_]^2–^ anions in an intermediate diameter (∼16 Å) SWCNT (Movie S3) (AVI)
Sequence showing the canonical motion of [Mo_6_Br_14_]^2–^ anions in a narrow ∼12 Å diameter SWCNT (Movie S4) (AVI)

## Supplementary Material

ja4c14883_si_001.pdf

ja4c14883_si_002.avi

ja4c14883_si_003.avi

ja4c14883_si_004.avi

ja4c14883_si_005.avi
