# Supplementary material for: Differential Packing of Cs2Mo6Br14 Cluster-Based Halide in Variable Diameter Carbon Nanotubes with Elimination and Polymerization to 1D [Mo2Br6]x Ising Model Structures by Steric Confinement
Source: J Am Chem Soc. 2025 Feb 19;147(9):7345–59. doi: 10.1021/jacs.4c14883 (PMC11887431; doi:10.1021/jacs.4c14883)
Supplement: Supplementary file 1 — ja4c14883_si_001.pdf [file ja4c14883_si_001.pdf]

Supplementary Information

Differential packing of $Cs_2Mo_6Br_{14}$ cluster-based halide in variable diameter carbon nanotubes with elimination and polymerization to $[Mo_2Br_6]_x$ 1D model Ising structures by steric confinement

Eric Faulques,[1*] *Victor G. Ivanov*[2], *Stéphane Cordier*[3], *Reza J. Kashtiban,*[4] *Yann Molard*[3], *Jean-Luc Duvail*[5], *Nataliya Kalashnyk*[1*], *Jeremy Sloan*[4*]

[1]Univ. Lille, CNRS, Centrale Lille, Univ. Polytechnique Hauts-de-France, UMR 8520 - IEMN, F-59000 Lille, France

[2]Sofia University, St. Kliment Ohridsky, Faculty of Physics, 5 James Bourchier Boulevard, 1164 Sofia, Bulgaria

[3] Univ Rennes, CNRS, ISCR, Institut des Sciences Chimiques de Rennes–UMR6226, F-35000 Rennes, France

[4]Department of Physics, University of Warwick, Coventry CV4 7AL, United Kingdom

[5] Nantes Université, CNRS, Institut des Matériaux de Nantes Jean Rouxel, IMN, F-44000 Nantes, France

Fig

Contents:

Table S1. Shannon and estimated ionic or atomic radii for Cs, Mo^{II}, Mo^{III}, Br^- and C^0.

Figure S1. $Cs_2Mo_6Br_{14}$ orientation scheme inside and outside a (10,10) SWCNT.

Figure S2 Raman spectra of cluster and cluster@SWeNT composite for $\lambda_{\varepsilon xc}$ = 647.1 nm.

List of descriptions of Movies S1 to S4

*To whom correspondence should be addressed. E-mail: eric.faulques@univ-lille.fr, nataliya.kalashnyk@univ-lille.fr, j.sloan@warwick.ac.uk

Table S1. | Shannon[48] and estimated ionic or atomic radii for Cs, Mo^{II}, Mo^{III}, Br^- and C^0. Electron configuration and coordination are also indicated.

Ion/Atom	Configuration	Coordination No.	Radii (Å)
Cs^+	$[Xe]6s^0$	-	1.67
Mo^{2+}	$[Kr]4d^45s^0$	6	0.607[†]
Mo^{3+}	$[Kr]4d^35s^0$	9	0.69
Br^-	$[Ar]3d^{10}4s^24p^6$	-	1.96
C^0	$[He]2s^22p^2$	3	1.7[‡]

†: Estimated from Mo-Br^a distance in $[Mo_2Br_6]_x$ polymer (i.e. 2.567Å less Br^- radius) based on the model in Figure 7(c).
‡: Carbon van der Waals radius.[S1]

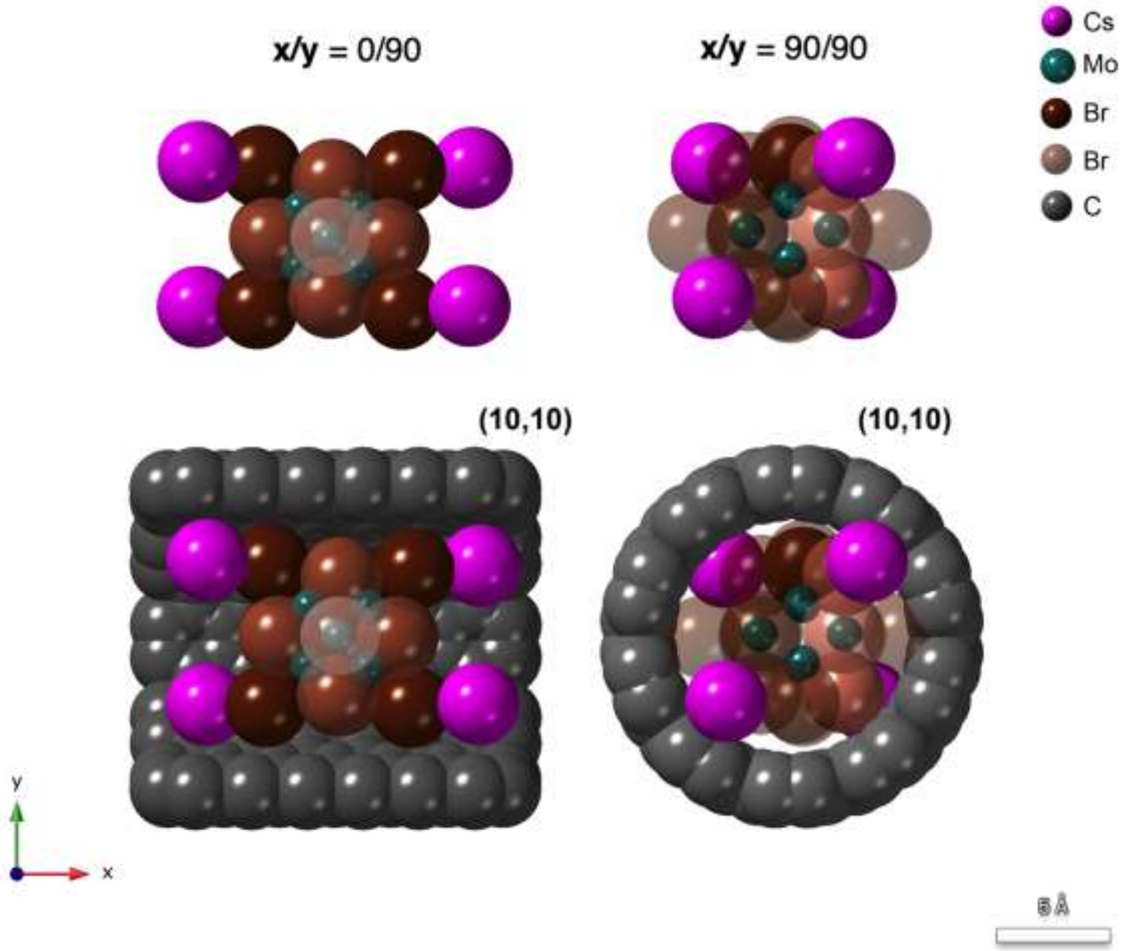

Figure S1. | $Cs_2Mo_6Br_{14}$ orientation scheme inside and outside a (10,10) SWCNT. Top: side on and end on views of unencapsulated $Cs_2Mo_6Br_{14}$. Bottom: side on and end on views of $Cs_2Mo_6Br_{14}$ cluster (with two extra Cs^+ ions) encapsulated within a (10,10) SWCNT.

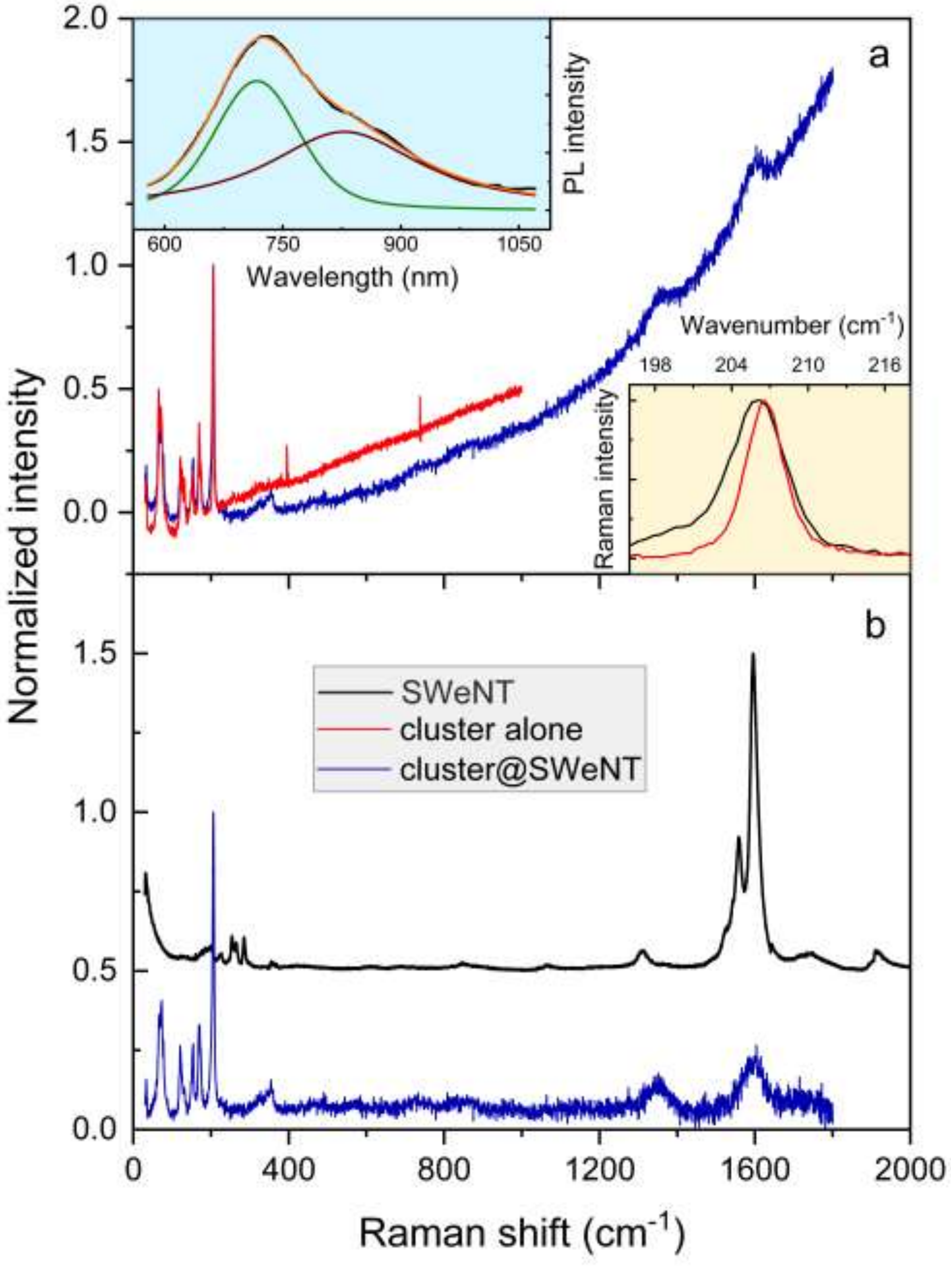

Figure S2. | Raman spectra of cluster and cluster@SWeNT composite for $\lambda_{\varepsilon xc}$ = 647.1 nm. Top panel (a): raw spectra of pristine cluster $Cs_2Mo_6Br_{14}$ crystallites (between 30 and 1000 cm^{-1} and cluster@SWeNT composite (between 30 and 1800 cm^{-1}). Inset left: photoluminescence spectrum of pristine cluster at room temperature and excitation at 510 nm. Inset right: Close-up of the strongest cluster line at 206 cm^{-1} showing a broadening after intercalation. Bottom panel (b): pristine SWeNT (upper curve) and cluster@SWeNT sample (lower curve)) with fluorescence background removed). In Table 1 (main article) SWCNT diameters were determined using $\lambda_{\varepsilon xc}$ = 633 nm and the relationship d(nm) = 234 / ω_{RBM}.[S2]

List of descriptions of Movies S1 to S4. All high resolution transmission electron microscopy (HRTEM) images were obtained at 80 kV acceleration in a JEOL ARM200F (full description Methods Section).

Movie S1 – Sequence showing vibration in position of the array of $[Mo_6Br_{14}]^{2-}$ anions featured in Fig. 1(e), main article. This representation shows the cluster array as viewed in the original micrographs. The animation consists of 14 images in a repeating loop and the anions are packed into a ~23 Å diameter SWCNT as depicted in Figs. 1(f) to (j). Cs^+ cations cannot be seen in this video.

Movie S2 – Sequence showing $[Mo_6Br_{14}]^{2-}$ anions packed into an outlier double walled carbon nanotube (DWCNT) in which the inner SWCNT is ~18 Å in diameter. Canonical motion of the $[Mo_6Br_{14}]^{2-}$ anions can be seen inside the inner SWCNT and motion of the anions can also be seen in several other nanotubes surrounding the DWCNT. Cs^+ cations cannot be seen in this video.

Movie S3 – Sequence showing motion of a ‘zig-zag’ array of $[Mo_6Br_{14}]^{2-}$ anions in an intermediate diameter (~16 Å) SWCNT. Both order and disorder in the zig-zag array can be seen in different parts of the sequence. Cs^+ cations cannot be seen in this video.

Movie S4 – Sequence showing canonical motion of $[Mo_6Br_{14}]^{2-}$ anions in a narrow ~12 Å diameter SWCNT. Cs^+ cations cannot be seen in this video.

References

S1. Bondi. A. van der Waals volumes and radii. *J. Phys. Chem.* **1964**, *68*, 441–451.

S2. Jorio, A.; Saito, R.; Hafner, J. H.; Lieber, C. M.; Hunter, M.; McClure, T.; Dressehaus, G.; Dresselhaus, M. S. Structural determination of isolated single-wall carbon nanotubes by resonant Raman scattering *Phys. Rev. Lett.* **2001**, *86*, 1118–1121.
